# Acute Severe Hyponatremia following Hysteroscopic Procedure in a Young Patient: A Case Report and Review of the Literature

**DOI:** 10.1155/2021/7195660

**Published:** 2021-09-20

**Authors:** Anwar S. Atieh, Omar K. Abu Shamma, Mohammad O. Abdelhafez, Muath A. Baniowda, Samia Abed, Basheer H. Babaa, Abdurrahman Hamadah, Kamel A. Gharaibeh

**Affiliations:** ^1^Al-Quds University, Faculty of Medicine, Jerusalem, State of Palestine; ^2^Hashemite University, Faculty of Medicine, Zarqa, Jordan

## Abstract

**Background:**

Hysteroscopic surgery is a minimally invasive procedure used to diagnose and treat intrauterine pathologies. It requires distension of the uterine cavity for the adequate visualization of the operative field. Glycine (1.5%) is one of the most commonly used solutions because it is nonconductive and also has good optical properties. However, acute hyponatremia is a critical complication that can develop after the absorption of a sufficient amount of the irrigation medium. *Case Presentation*. We report a case of a 43-year-old female patient who developed acute symptomatic hyponatremia (104 mEq/L) and pulmonary edema secondary to hysteroscopic resection of leiomyoma and hastily approached with rapid sodium correction measures.

**Conclusion:**

Multiple strategies can be taken to reduce the risk of fluid absorption and subsequent hyponatremia. Moreover, attention should be paid to the treatment approach for patients with acute hyponatremia following hysteroscopic procedures; rapid correction of acute hyponatremia for such patients might be safe, although there is no consensus in the literature, and further trials are needed.

## 1. Background

Hysteroscopy has implemented a minimally invasive procedure for treating and diagnosing intrauterine pathology. A uterine distending medium is used to allow an optimal and global view of the endometrial cavity. Nonconductive (i.e., nonelectrolyte) and conductive solutions are widely used as a distension medium for hysteroscopic procedures. Glycine is one of the most commonly used irrigation fluids in hysteroscopy; it is a hypoosmotic solution compared to serum osmolality, and it has an osmolality of 200 mosmol/kg [1].

Complications from hysteroscopy are rare, with a reported rate of 0.22%. Hyponatremia and fluid overload are considered as feared but rare complications of hysteroscopy; according to a multicenter study of over 21,000 operative hysteroscopic procedures, the incidence rate of fluid overload and hyponatremia is 0.06% [2]. The volume of medium absorbed, which is measured by volume deficit, is the most significant risk factor for the development of hyponatremia, and it has a direct relationship with the degree of hyponatremia [3].

Patients with acute hyponatremia due to hysteroscopy can be asymptomatic. They can also have variable symptoms, such as nausea, vomiting, confusion, and convulsions, depending on the severity of hyponatremia and irrigant absorption rate. Furthermore, the use of general anesthesia can also conceal the symptoms of hyponatremia [4–6].

Hyponatremia in hysteroscopic procedures has been reported with the use of 1.5% glycine, 3% sorbitol, 5% mannitol, and 5% dextrose which are the major nonconductive (i.e., nonelectrolyte) irrigation and distension fluids [7–10].

A number of factors should be considered when selecting distention media for hysteroscopy, including the procedure to be performed and the instruments to be used. The traditional electrosurgery devices have a monopolar design and can be only used with electrolyte-free irrigation fluids [11]. However, bipolar electrosurgery devices are now more widely used.

Here, we report on a patient undergoing a hysteroscopic removal of leiomyoma with an endometrial ablation, who developed acute symptomatic hyponatremia (104 mEq/L) due to the irrigation fluid (1.5% glycine).

## 2. Case Presentation

A 43-year-old woman (weight, 70 kg) presented with a history of 7-month duration of intermittent and prolonged vaginal bleeding. A physical examination and an ultrasound showed a submucosal leiomyoma that protruded into the uterine cavity. There were no other symptoms or pathological findings. The patient had a free past medical and surgical history. She had no known allergies. She was taking daily doses of ethinyl estradiol (0.03 mg) and drospirenone (3 mg) continuously over 3 months and iron bisglycinate (36 mg) over the same period. The patient was offered scheduled hysteroscopic removal of the leiomyoma, and an endometrial ablation under general anesthesia (GA) was decided on, after which the patient gave written informed consent.

Preoperative laboratory findings, which included a complete blood exam (CBC), electrolytes analyses, coagulation parameters, showed anemia with hemoglobin of 7.5 g/dL, and a 12-lead electrocardiogram (ECG) showing sinus tachycardia. Most notably, both creatinine and sodium were within normal levels, that is, 1.1 mg/dL and 139 mEq/L, respectively. Upon arrival at the operating room, the patient was placed in the lithotomy position followed by the application of a peripheral venous catheter and standard monitoring devices (ECG, noninvasive blood pressure cuff (NIBP), and pulse oximetry (SpO_2_)). Immediately before the anesthesia induction, vital signs were blood pressure 104/69 mmHg, heart rate 107 beats/min, pulse oximetry revealed 100% of SpO_2_, and temperature of 37.6°C before anesthetic induction; general anesthesia was induced intravenously with propofol and sufentanil. An endotracheal tube (ETT) was facilitated with rocuronium, and after the correct positioning was verified, the lungs were ventilated with a fraction of inspired oxygen (FiO_2_) of 0.5 and a minute volume (MV) of 74 mL/min/kg. After anesthesiological clearance, the hysteroscopy commenced, dilation was done by Hegar dilator till #9. Anesthesia was maintained with continuous infusion of propofol. Glycine (1.5%) solution was used as the distension medium. This standard hypotonic solution contains 1.5 g glycine per 100 mL of water and has an osmolality of 200. A pressure of 120 mmHg was applied to unfold the uterine cavity. Endometrial ablation and dissection of a 2.5*∗*3*∗*3 cm submucosal fundal myoma were performed within 20 min by monopolar resection. The patient remained in a hemodynamically stable condition throughout the procedure.

The ET tube was removed, and the patient was immediately sent to the recovery area. Upon recovery, the SpO_2_ level suddenly fell from 98% to 80% to 85%, and supplemental O_2_ at a rate of 3 liters per minute by nasal cannula was applied and immediately treated with 20 mg of furosemide. Her chest auscultation was clear with a good air entry bilateral, and her blood pressure was 134/72, SpO_2_ 90%, and pulse of 56 beats per minute (b/m), abdominal ultrasound was free, and vaginal examination revealed no active bleeding. The bladder was catheterized, with a urine output of 2500 mL for a duration of 30 mins, and the patient was sent to the gynecological ward. Five minutes later, the patient showed desaturation of SpO_2_ 85%, became drossy, and developed blurred vision. Supplemental O_2_ at a rate of 5 Liters per minute by face mask was applied, 20 mg of furosemide was given, and blood tests and electrolyte analyses indicated a drop of hemoglobin to 6 g/dL and hyponatremia (Na 104 mEq/L). About 500 mL of 3% saline solution was immediately administered at an infusion rate of 500 mL/h and 2 units of PRBCs were given with precisely monitoring for electrolyte changes every hour. Meanwhile, the patient started complaining of headaches, nausea, and vomiting. One hour later, serum electrolyte analyses showed an elevation of Na level to 118. The order was given to switch fluids to normal saline with a rate of 100 cc/h, repeat serum analysis every hour, and do a neurological exam every 30 minutes. Plasma sodium levels increased to 119 mEq/L after 1 h and to 134 mEq/L after 6 h, as shown in Table 1.

The neurological assessment revealed no detectable deficits, and symptoms were revealed except for a headache. 12 hours after the onset of hyponatremia, the patient was off oxygen, and SpO_2_ was 98% on room air, no complaints, and sodium level was within normal (Na 135 mEq/L). Until then, the cumulative renal excretion was 8.5 L. 9 liters of distension medium was used intraoperatively with a calculated uptake of 4 L. A sample was not obtained for serum osmolarity determination. The patient recovered uneventfully and was discharged from the hospital on the second postoperative day. During a 6-month follow-up, the patient remained asymptomatic with no neurologic complications.

## 3. Discussion

Fluid overload and hyponatremia are life-threatening complications associated with the use of nonconductive hypoosomolar distension media in hysteroscopy. Sufficient absorption of solutions like 1.5% glycine, 3% sorbitol, and 5% mannitol is more associated with dilutional hyponatremia, whereas using isotonic normal saline is associated with fluid overload [12]. Fluid absorption into the vascular space (intravasation) is the central underlying mechanism for fluid overload and hyponatremia problems. The main driving force for intravasation is the distension media pressure. In 250 operative hysteroscopies, there was no significant fluid absorption when keeping the intrauterine pressure below 80 mmHg [13]. Furthermore, procedures that invade the myometrium expose the uterine vascular system, which increases the potential for fluid intravasation [14].

The data regarding the rule of type of anesthesia on fluid absorption are conflicting; a randomized prospective comparative study by Goldenberg et al. found that fluid absorption is significantly higher with hysteroscopies done by epidural anesthesia when compared to general anesthesia. They hypothesized that epidural anesthesia causes vasodilation of the pelvic vessels, a consequence caused by the known effect of epidural anesthesia in sympathetic blocking [15, 16]. Conversely, Goldenberg et al. did not study the impact of local anesthesia with intravenous sedation on fluid distention media absorption. On the contrary, a retrospective cohort study by Bergeron et al. found that fluid absorption was the highest with general anesthesia and the lowest when using local anesthesia with intravenous sedation, they believe that inhaled anesthetics used in general anesthesia have a potent effect on the absorption of glycine. Inhaled anesthetics dilate arteriolar muscles, thus inducing vasodilatation, which leads to the intravasation of distension fluid. Moreover, a higher rate of glycine fluid absorption and a more rapid decrease in serum sodium concentration were associated with general anesthesia. Thus, they recommend considering regional anesthesia with sedation for hysteroscopic procedures using glycine as a distension medium [17, 18].

In a randomized trial of 33 women who had a hysteroscopic endometrial ablation, administration of intracervical vasopressin solution (0.05 U/mL) was associated with a significant decrease in plasma glycine concentration but a small nonsignificant reduction of dilutional hyponatremia [19]. In another small prospective series comprising 36 patients, who underwent transurethral resection of the prostate (TURP), the use of intraprostatic vasopressin has possible protection against hyponatremia. They presume that vasopressin constricts the prostatic blood vessels and reduces bleeding, which in turn enhances the visibility of the prostatic capsule and allows early identification of capsular tear, as well it could decrease irrigant fluid absorption [20]. In another prospective controlled study, 88 patients underwent hysteroscopic procedures. They used general anesthesia in 62 cases (control group), whereas spinal anesthesia combined with oxytocin infusion was used in the other 26 cases (study group). They presumed that using low-dose oxytocin (10 international units (IUs) in 200 mL normal saline) would stimulate uterine contractions compressing the myometrial vessels and reduce the glycine absorption. There was a statistically significant less mean fluid deficit in the study group than the control group in endometrial polypectomy and myomectomy patients but not in those who underwent septal resection [21].

The 1.5% glycine is one of the most common solutions used in monopolar hysteroscopic procedures due to its nonhemolytic, nonconductive, and transparent properties, although it is more toxic and has poorer outcomes when confronted with other options [22]. The absorption of glycine solution into the extracellular space will make the blood hypertonic with a higher volume, which will draw the fluid outside the cells. Both events, in turn, will cause hyponatremia due to the dilutional effect. A few hours later, the glycine will be absorbed by cells and metabolized into ammonia [23].

The severity of hyponatremia is highly connected to the volume deficit of the glycine solution; a volume deficit of 1000 mL will reduce sodium concentration in the plasma by 10 mEq/L [3]. An excessive amount of fluid absorption is not only associated with hyponatremia; it may develop to cause pulmonary edema that can present with hypoxia and shortness of breath [24].

Moderate to severe hyponatremia, associated with glycine irrigant absorption, can cause many serious neurological symptoms like seizures, confusion, coma, and visual disturbances [25]. Visual disturbances were linked to the glycine inhibitory effect on retinal cells [26]. However, hyponatremic encephalopathy does not have a specific neurologic presentation but is mainly present with headache, nausea, and vomiting [27]. The severity of these complications determined mainly by the presence of cerebral edema and hypoosmolality [28]. Most publications reported neurological or respiratory presenting symptoms as a complication of hyponatremia or fluid overload associated with hysteroscopic procedures as shown in Table 2. Ayus-Arieff syndrome is a common but unrecognized complication of severe hyponatremia where cerebral edema drives neurogenic pulmonary edema. This phenomenon was noticed among healthy marathon runners who presented with hyponatremia secondary to dehydration and developed pulmonary edema that frequently requires mechanical ventilation [29]. The underlying mechanism of this syndrome is that cerebral edema leads to increased intracranial pressure, leading to centrally mediated increases in catecholamine release and capillary injury. The pulmonary vasculature seems to be the most affected. In addition, pulmonary artery hypertension and plasma leakage through the injured capillaries cause pulmonary edema and, as a sequel, acute respiratory distress syndrome [30]. For our presented patient, rapid correction of the hyponatremia led to clearing the cerebral edema and, consequently, neurogenic pulmonary edema.

Hyponatremia caused by using glycine solution has a less pronounced fall in the measured plasma osmolality due to the presence of osmotically active particles of glycine in extracellular space. Although there is a modest reduction in serum osmolality, yet marked neurologic symptoms still occur [23, 44]. This can be explained by the presence of ammonia as a secondary metabolite of glycine and the toxicity from glycine itself since it has a neuroinhibitory effect on the central nervous system [26, 45]. Also, it is found that cerebral edema was more frequent in premenopausal women, which could be attributed to the suppression of Na+/K+ ATPase pump caused by female sex steroids; these pumps normally decrease the risk of cerebral edema caused by hyponatremia [27].

It is well established that distending media containing electrolytes urge the use of bipolar resectoscope instruments. These newer devices are compatible with electrolyte-containing irrigation and distension solutions such as isotonic saline and Ringer's lactate. The use of isotonic saline or Ringer's lactate solutions as a distension medium is not associated with hyponatremia or hypoosmolality [1, 46]. On the contrary, the use of monopolar instrumentation requires electrolyte-free solutions such as 5% mannitol, 1.5% glycine, and 3% sorbitol, to facilitate the completion of the radiofrequency electrical circuit [47]. Hence, hyponatremia should always be considered following a unipolar hysteroscopic procedure. Multiple measures can be taken to avoid it. Replacement of unipolar electrosurgery by bipolar significantly reduces the risk. A meta-analysis study showed sodium mean decrease of 1.5 mEq/L when using bipolar electrosurgery comparing to unipolar electrosurgery (5.1 mEq/L) [48, 49]. Furthermore, reduction of the procedure time is an important factor for the development of hyponatremia since longer procedure duration is associated with a higher absorption deficit. The *American College of Obstetricians and Gynecologists* (ACOG) suggests to temporarily halt the procedure after 750 mL absorption deficit and termination of the procedure if the deficit exceeds 1000 mL [49]. Careful monitoring of fluid in and out during the hysteroscopy will help the surgeon determine when to stop the procedure before excessive fluid absorption elicits a symptomatic hyponatremia or fluid overload [37].

The treatment of excessive fluid absorption and subsequent hyponatremia depends on the patient's clinical picture. Asymptomatic hyponatremia does not require any urgent intervention, whereas marked, symptomatic hyponatremia must be treated as quickly as possible to reduce the risk of complications. Rapid correction of acute hyponatremia is probably safe, as the cerebral adaptation needs time to take place [50]. Based on Ayus et al. study, rapid infusion of hypertonic saline is recommended for correcting severe acute hyponatremia [23], and our patient was managed accordingly. However, her sodium level rapidly increased from 104 mEq/L to 118 mEq/L in 1 h. A series study of 64 hyponatremic encephalopathy patients presented to the emergency department was treated with a continuous intravenous infusion of 500 mL of 3% sodium chloride solution over 6 h. Although 12 deaths were reported, all of which were recorded after the resolution of hyponatremic encephalopathy and were postulated to comorbid conditions, 75% of deaths were related to sepsis. They concluded that 3% sodium chloride solution was effective in reversing neurologic symptoms, and clinical evidence of cerebral demyelination, permanent neurologic injury, or death within 6 months posttreatment follow-up [51].

Unfortunately, there is no evidence from controlled trials, as to what the best or optimal correction rate is in acute hyponatremia following hysteroscopic procedures.

Nevertheless, ACOG guidelines recommended that the serum sodium level should not be raised more than 12 mEq/L in the first 24 h with strict monitoring; to prevent reaching normal or hypernatremic levels [52–54]. ACOG also concluded that the rate of correction should be slower (8–10 mEq/L maximum increase in the first 24 h) in patients who present with hyponatremia more than 48 h after the procedure [54]. A hypertonic saline infusion may play a substantial role by reduction of cerebral edema and replacement of sodium. A series of 18 patients underwent TURP or hysteroscopy; Fourteen of them developed hyponatremia, they were treated supportively, and received hypertonic saline. They survived without long-term sequelae. Two males suffered respiratory arrest as the initial clinical manifestation of hyponatremic encephalopathy; both patients died and did not receive hypertonic saline. Two other female patients, who did not survive, recovered from anesthesia but had nausea, emesis, and headache, and both experienced respiratory arrests. After the respiratory arrest, their serum sodium levels were 125 and 120 mmol/L; both patients died, and autopsies revealed cerebral edema with tonsillar herniation. However, the degree of hyponatremia or the amount of fluid retention was not the determinant factor for the four deaths. This retrospective study cannot be conclusive, and other factors might play a role in determining morbidity and mortality [23].

When the hyponatremia is associated with a relatively normal measured serum osmolality, in such cases where glycine remains confined to the extracellular fluid, hemodialysis should be considered since hypertonic saline therapy is associated with an increased risk of pontine myelinolysis. Hemodialysis showed to safely stabilize the patients and correct the electrolyte and fluid disturbances, and it gives the benefits of correcting the hyponatremia while eliminating the unmeasured solute [14, 44]. Indeed, it is hard to control the correction rate, and it is often more rapid than expected. In our case, the patient received a total of 500 mL of 3% hypertonic saline in 1 h. The correction rate for hyponatremia was faster than expected, and the serum sodium level spontaneously increased to 135 mEq/L within 14 h. She recovered uneventfully and remained asymptomatic with no neurologic complications after 6 month on follow-up. Our presented case is in agreement with a number of reported cases that have shown severe hyponatremia during hysteroscopic procedures, provided in Table 2. None of these publications reported a detrimental effect associated with the rapid correction of hyponatremia. Furthermore, all patients recovered fully, even in cases with very low sodium levels.

## 4. Conclusion

The systemic absorption of distending medium used in hysteroscopic producers can have multiple complications, of which acute hyponatremia can have severe consequences. Therefore, multiple measures can be taken to avoid those complications. Using electrolyte-based distending media instead of hypoosmolar solutions significantly reduces the risk. In addition, attention should be on reducing and precisely monitoring fluid intake. Furthermore, some studies have focused on the role of anesthetic technique on fluid absorption, using local anesthesia instead of other types, combining intravenous oxytocin infusion with spinal anesthesia, or the concomitant use of intrauterine vasopressin revealed promising protection against volume overload and hyponatremia. Future randomized trials ought to focus on the impact of anesthesia and such medications on developing hyponatremia. Besides, we should review the role and the outcome of rapid correction of acute hyponatremia in such cases, since anecdotal reports are not in line with the literature regarding developing osmotic demyelinating syndrome. Conclusively, our case supports the advantage of the rapid correction of acute hyponatremia.

## Figures and Tables

**Table 1:** The patient's electrolyte data.

	Sodium (mEq/L)	Potassium (mEq/L)
Preoperatively		
Before the procedure	139	3.8

Postoperatively		
10 minutes after the procedure	104	3.5
1 hour	118	3.7
2 hours	119	3.7
8 hours	134	3.5
12 hours	135	3.7

**Table 2:** Overview of reported cases with acute symptomatic hyponatremia during hysteroscopic procedures.

Case report	Lowest Na+ level, mmol/L	Normal plasma Na+ after, h	Est. fluid absorption, L	Irrigation fluid used	Max. fluid pressure, mmHg	Surgery time, min	Treatment for hyponatremia	First clinical manifestation	Presence of pulmonary edema	Type of anesthesia	Discharge day
Okazaki et al. [31]	84.1	15	7	3% d-sorbitol solution	N/A	123	Furosemide; 1.5% saline; acetate Ringer	Edemas in the conjunctiva, lip, and fingers	Yes	GA	6

Palanisamy et al. [32]	125	N/A	N/A	1.5% glycine	100	120	Furosemide	Abdominal distension and facial puffiness	No	GA	N/A

Liao et al. [33]	125	3	N/A	5% dextrose water	N/A	N/A	Sodium bicarbonate, 3% NaCl; mannitol; furosemide	Postoperative cardiac arrest	Yes	GA	28

Elegante et al. [34]	125	6	2.7	1.5% glycine	N/A	30	Furosemide	Hypoxia; dyspnea and altered mental status	Yes	N/A	Day 2

Chauha et al. [35]	111	6	N/A	1.5% glycine	90	120	Furosemide	Swelling of the face and vision problem	No	GA	Day 1

Sultan [36]	119	3	1.2	1.5% glycine	100	65	Furosemide	Confusion	No	Spinal anesthesia	Day 2

Hepp et al. [37]	74	7	5	2.7% sorbitol and 0.54% mannitol	120	70	3% NaCl; furosemide	Dyspnea	Yes	GA	Day 3

Almonti et al. [38]	120	10	2	1.5% glycine	N/A	60	7% NaCl; furosemide; mannitol	Myoclonus	No	GA	Day 2

Yaprak et al. [9]	99	14	5	Mannitol 5%	N/A	N/A	3% NaCl; furosemide	Altered mental status and agitation	No	N/A	Day 2

Sethi et al. [7]	100	N/A	1	1.5% glycine	80	45	3% NaCl; furosemide; sodium bicarbonate	Hypoxia	Yes	GA	Day 14

Yang and Feng [10]	120	12	3	Dextrose 5%	150	70	3% NaCl; furosemide	Flatulence and coma	No	N/A	N/A

Woo et al. [8]	87	48	24	2.7% sorbitol and 0.54% mannitol	150	60	3% NaCl; furosemide	Dyspnea	Yes	GA	Day 7

Wegmuller et al. [39]	78	36	N/A	2.7% sorbitol and 0.54% mannitol	100	80	Furosemide; hypertonic NaCl; bicarbonate 8.4%	Desaturation and laryngeal edema	Yes	Spinal anesthesia	Day 30

Jo et al. [5]	83	24	N/A	5:1 sorbitol/mannitol	120	150	Furosemide; 3% NaCl	Hypoxia	Yes	GA	Day 4

Serocki et al. [40]	106	24	0.4 L–4	2.7% sorbitol and 0.54% mannitol	180	25	0.9% NaCl	Hypoxia	Yes	GA	Day 2

Lee et al. [41]	89	24	7	2.7% sorbitol and 0.54% mannitol	100	40	2% NaCl; sodium bicarbonate; furosemide;	Hypotonia and bleeding leading to hysterectomy	No	GA	Day 2

Estes and Maye [42]	122	8	2.4	1.5% glycine	N/A	90	Furosemide	N/A	No	GA	Day 1

Arieff and Ayus [43]	137	N/A	N/A	3% sorbitol and 1.5% glycine	N/A	156 ± 24	Hypertonic (514 mmol/L) sodium chloride	Tremulousness, hypothermia, hypoxia, postoperative: Headache, nausea, emesis, and seizures	No	GA	N/A

## Data Availability

The plasma sodium level data used to support the findings of this study are included within the article. Any further data are available from the corresponding author upon request.
